# Supplementary figures and images for: Biology and ecology of the lionfish Pterois volitans/Pterois miles as invasive alien species: a review
Source: PeerJ. 2023 Jul 25;11:e15728. doi: 10.7717/peerj.15728 (PMC10377442; doi:10.7717/peerj.15728)

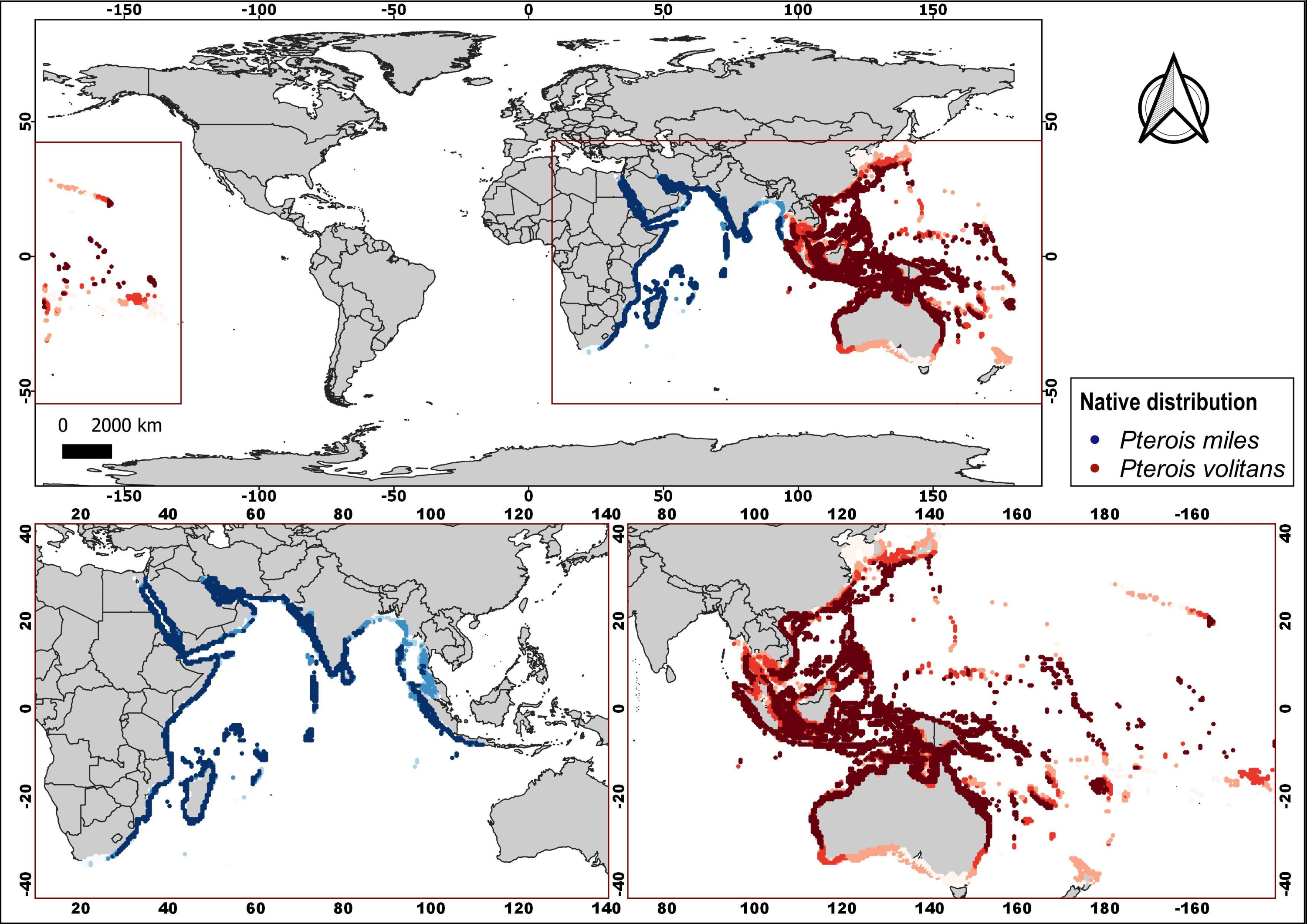

Supplement: Supplemental Information 1 — Dark colors are higher probabilities of occurrence. Maps were built based on information from FishBase (AquaMaps: Kaschner et al. (2019)). [file peerj-11-15728-s001.png]
