# Supplementary material for: Biology and ecology of the lionfish Pterois volitans/Pterois miles as invasive alien species: a review
Source: PeerJ. 2023 Jul 25;11:e15728. doi: 10.7717/peerj.15728 (PMC10377442; doi:10.7717/peerj.15728)
Supplement: Supplemental Information 2 [file peerj-11-15728-s002.docx]

**Biology and ecology of the lionfish *Pterois volitans / Pterois miles* as invasive alien species: a review**

Laura del Río^1^, Zenaida Navarro-Martínez^1^, Dorka Cobián Rojas^2^, Pedro Chevalier Monteagudo^3^, Jorge Angulo-Valdes^4^, Leandro Rodríguez-Viera^1*^

^1^Center for Marine Research, University of Havana, 16 street No. 114 between 1st and 3rd, Miramar, Playa, CP 11300, Havana, Cuba

^2^Guanahacabibes National Park, Center of Research and Evironmental Services, Ministry of Science, Technology and Environment, La Bajada, 22100 Pinar del Río, Cuba.

^3^National Aquarium of Cuba. Calle 1^st^ street No. 6002, 11300 Playa, Havana, Cuba.

^4^Eckerd College, 4200 54th Ave S, St. Petersburg, FL 33711, USA

* Corresponding author (leokarma@gmail.com)

Table S1. Most relevant lionfish studies along the invasive and natural distribution ranges, which were used along this review.

| **Topic** | **Country/Region** | | **Locality** | **Reference** |
| --- | --- | --- | --- | --- |
| **Diet** |  | |  |  |
|  | Bahamas | |  | Morris and Akins (2009) |
|  | United States | | North Carolina | Muñoz *et al*. (2011) |
|  | Bonaire | |  | McCleery (2011) |
|  | Mexico | | Cozumel, Xcalak, Mahahual, Banco Chinchorro, Puerto Morelos, Playa del Carmen, Isla Mujeres | Valdez-Moreno *et al*. (2012) |
|  | Gulf of Mexico | |  | Dahl and Patterson III (2014) |
|  | Mexico | | Quintana Roo | Villaseñor-Derbez and Herrera-Pérez (2014) |
|  | Cuba | | Holguín | Vega *et al*. (2015) |
|  | Cuba | | Guanahacabibes National Park | Cobián-Rojas *et al*. (2016) |
|  | Mexico | | Mexican Caribbean | Arredondo-Chávez *et al*. (2016) |
|  | Cuba | | Guanahacabibes, La Habana, Cayo Las Brujas | Pantoja *et al*. (2017) |
|  | Northern Gulf of Mexico | |  | Dahl *et al.* (2017) |
|  | Bermuda | |  | Peake *et al.* (2018) |
|  | United States | | Biscayne National Park | Sancho *et al*. (2018) |
|  | Bermuda | |  | Eddy (2019) |
|  | Bermuda | |  | Eddy *et al.* (2020) |
|  | Curaçao | |  | Ritger *et al.* (2020) |
|  | Mexico | | Veracruz | Aguilar-Medrano and Vega-Cendejas (2020) |
|  | Mexico | | Cozumel | Bogdanoff *et al*. (2020a) |
|  | United States | | Western Florida | Santamaria *et al*. (2020) |
| **Behavior** |  | |  |  |
|  | Israel | | Eilat | Fishelson (1997) |
|  | Bahamas | | New Providence | Coté and Maljkovic (2010) |
|  | Bahamas | | Eleuthera | Green *et al*. (2011) |
|  | Philippines,Guam and Cayman Islands | |  | Cure *et al*. (2012) |
|  | Bahamas | | Lee Stocking Island | Albins and Lyons (2012) |
|  | Bahamas | | Rock Sound,  west of Cape Eleuthera | Tamburello and Cote (2014) |
|  | Mexico | | Mahahual, Banco Chinchorro Biosphere Reserve, Xcalak Reef National Park | García-Rivas *et al.* (2017) |
|  | Curaçao | |  | Ritger *et al.* (2020) |
|  | United States | |  | Bogdanoff *et al.* (2020b) |
|  | United States | | Florida | Dahl and Patterson III (2020) |
| **Invasion** |  | |  |  |
|  | United States, Bermuda | | United States: North Carolina, New York, Georgia, Florida | Whitfield *et al*. (2002) |
|  | Dominican Republic | | Morro (La Piedra de Kikim) | Guerrero and Franco (2008) |
|  | Cuba | |  | Chevalier *et al*. (2008) |
|  | United States, Turks and Caicos Islands, Bahamas, Bermuda, Cayman Islands, Cuba, Jamaica, Dominican Republic, Puerto Rico, Mexico, Honduras, Costa Rica | |  | Schofield (2009) * |
|  | United States, Bermuda, Bahamas, Islas Turks and Caicos, Cayman Islands, Cuba, Jamaica, Dominican Republic, Puerto Rico, Mexico, Haiti, Nicaragua, Colombia, Honduras, Costa Rica, Panamá, Venezuela, Leeward Islands | |  | Schofield (2010) * |
|  | Bermuda, Cayman Islands, Colombia | | Cayman Islands: Grand Cayman, Colombia: San Andrés Archipelago, Santa Marta | Betancur-R. *et al*. (2011) |
|  | United States | | Florida Key | Ruttenberg *et al*. (2012) |
|  | Brazil | | Arraial do Cabo | Ferreira *et al*. (2015) |
|  | United States | | Coastal waters of the Gulf of Mexico and Atlantic Ocean | Lyons *et al*. (2020) |
| **Abundance** |  | |  |  |
|  | Taiwan | |  | Chen *et al.* (2004) |
|  | Australia | | Middleton and Elizabeth Reef | Oxley *et al.* (2004) |
|  | Western north Atlantic | |  | Whitfield *et al*. (2006) |
|  | Bahamas | | New Providence | Green and Coté (2009) |
|  | Kenya, Bahamas | | Bahamas: New Providence | Darling *et al*. (2011) |
|  | Costa Rica | |  | Sandel (2011) |
|  | Indo-Pacific | |  | Kulbicki *et al*. (2012) * |
|  | Bahamas | | New Providence | Green *et al*. (2012) |
|  | United States | | Florida Key | Ruttenberg *et al*. (2012) |
|  | Cuba, Bahamas, Mesoamerican Barrier Reef in Belize and Mexico | | Cuba: Jardines de Reina | Hackerott *et al*. (2013) |
|  | Curaçao, Bonaire | |  | de León *et al*. (2013) |
|  | Gulf of Mexico | | North of the Gulf of Mexico | Dahl and Patterson III (2014) |
|  | Bahamas | | Island of San Salvador | Anton *et al*. (2014) |
|  | Venezuela | | Morrocoy national park, Ocumare de la Costa, Chichiriviche de la Costa, Chuspa, Mochima National Park | Agudo and Klein (2014) |
|  | Cuba | | Guanahacabibes National Park | Cobián-Rojas *et al.* (2016) |
|  | Cuba | |  | Chevalier (2017) |
|  | Belize | | Bacalar Chico Marine Reserve | Anderson *et al*. (2017) |
|  | Cuba | | San Felipe National Park | Guardia *et al.*, 2017 |
|  | Bermuda | |  | Goodbody-Gringley *et al*. (2019) |
|  | Gulf of Mexico | |  | Dahl *et al.* (2019) |
|  | Mexico | | Veracruz | Aguilar-Medrano and Vega-Cendejas (2020) |
| **Impact** |  | |  |  |
|  | Bahamas | | Lee Stocking Island | Albins and Hixon (2008) |
|  | Bahamas | | New Providence | Green *et al.* (2012) |
|  | Bahamas | | Lee Stocking Island | Albins (2013) |
|  | Bahamas | | Lee Stocking Island | Albins (2015) |
|  | Venezuela | | Archipelago Los Roques National Park | Elise *et al.* (2015) |
|  | Southeastern United States, Caribbean, Gulf of Mexico | |  | Ballew *et al.* (2016) |
|  | Bahamas | | Eleuthera | Eaton *et al.*, 2016 |
|  | Bahamas | | Rock Sound | Tuttle (2017) |
|  | Bahamas | | Stocking Island | Kindinger and Albins (2017) |
|  | Cuba | | Guanahacabibes, La Habana, Cayo Las Brujas | Pantoja *et al*. (2017) |
|  | Bahamas | | Great Bahama Bank | Kindinger and Albins (2017) |
|  | United States | | Biscayne National Park | Curtis *et al.* (2017) |
|  | Colombia | | Santa Marta, Capurgana, Isla de San Andrés, Cabo de la Vela | Rojas-Vélez *et al.* (2019) |
|  | Cuba, Mexico | | Cuba: Guanahacabibes, Mexico: Arrecifes de Xcalak | Cobián-Rojas *et al.* (2018a) |
|  | Northern Gulf of Mexico | |  | Chagaris *et al*. (2020) |
|  | Honduras | | La Ensenada, Tela Bay | Hunt *et al*. (2020) |
|  | Mexico | | Xcalak | Murillo-Pérez *et al*. (2021) |
| **Control** |  | |  |  |
|  | Bahamas | | New Providence, Eleuthera | Maljkovic and Van Leeuwen (2008) |
|  | Curaçao, Bonaire | |  | de León *et al*. (2013) |
|  | Cuba, Bahamas, Mesoamerican Barrier Reef in Belize and Mexico | | Cuba: Jardines de Reina | Hackerott *et al*. (2013) |
|  | Belize | | Bacalar Chico Marine Reserve (BCMR) | Anderson *et al*. (2017) |
|  | Gulf of Mexico | |  | Harris *et al*. (2019) |
|  | Bahamas | | Eleuthera | Tamburello *et al.* (2019) |
|  | Mexico | | Cozumel Island | Chel-Guerrero *et al*. (2020) |
|  | Belize; Cayman Islands | | Cayman Islands : Little Cayman Island | Bogdanoff *et al.* (2020c) |
|  | United States; Bahamas | | United States : North Carolina | Bogdanoff *et al*. (2020d) |
|  | United States: Virgin Islands | |  | Simnitt *et al.,* 2020 |
|  |  | |  |  |
| **Genetic/Phylogenetic** |  | |  |  |
|  | Jordania, Kenya, Sri Lanka, Indonesia, Taiwan | |  | Kochzius *et al*. (2003) |
|  | Indo-Pacific  United States | | United States:  North Carolina, New York, Florida | Hamner *et al*. (2007) |
|  | Cuba | | Guanahacabibes | Labastida *et al.* (2015) |
|  | Mexico | | Chinchorro Bank | Guzmán-Méndez *et al*. (2017) |
|  | Turkey, Cyprus, Israel, Jordania, South Africa Madagascar, India, Sri Lanka, Indonesia, Japan | |  | Stern *et al*. (2018) |
|  | Mexico, Cuba, Belize, Puerto Rico | | Mexico; Banco Chinchorro Biosphere Reserve, Puerto Morelos Reef National Park, Xcalak National Reef Park, Veracruz Coral Reef System National Park; Cuba: Guanahacabibes Biosphere Reserve; Belize: Turneffe Atoll Marine Reserve; Puerto Rico: La Parguera | Labastida-Estrada *et al.*, 2019 |
|  | Bahamas, Mexico, Belize, Honduras, Panamá, Filipinas, Taiwan, Sri Lanka | |  | Burford *et al*. (2019) |
| **Toxicology** |  | |  |  |
|  | Islas Marshall | |  | Saunders and Taylor (1959) |
|  | United States | | San Francisco | Cohen and Olek (1989) |
|  | Cuba | |  | Squadrone *et al*. (2019) |
|  | United States | | Florida | van den Hurk *et al*. (2020) |
| **Reproduction/Development** | |  |  |  |
| **Reproduction** | |  |  |  |
|  | United States, Bahamas | | United States: North Carolina | Morris *et al*. (2011a) |
|  | Cayman Islands | | Little Cayman Island | Gardner *et al*. (2015) |
|  | Cuba | | Havana | Cruz-López *et al.* (2020) |
| **Larval duration** |  | |  |  |
|  | Bahamas | |  | Ahrenholz and Morris (2010) |
| **Crecimiento** |  | |  |  |
|  | Colombian Caribbean | |  | Bustos-Montes *et al*. (2020) |

*Artículos no incluidos en el mapa

**Literature Cited**

Agudo, E.A., and E. Klein Salas. 2014. Lionfish abundance, size structure and spatial distribution along the Venezuelan coast (*Pterois volitans*, Pteroinae: Scorpaenidae). Rev. Biol. Trop. 62 (3): 151-158. http://www.scielo.sa.cr/scielo.php?script=sci_arttext&pid=S0034-77442014000700025&lng=en&nrm=iso

Aguilar-Medrano, R., and Ma.E. Vega-Cendejas. 2020. Size, weight, and diet of the invasive lionfish *Pterois volitans* (Linnaeus, 1758) on the southern coast of Veracruz, Gulf of Mexico. Ciencias Marinas 46(1):57–64. https://doi.org/10.7773/cm.v46i1.3012.

Ahrenholz, D.W., and J.A. Morris. 2010. Larval duration of the lionfish, *Pterois volitans* along the Bahamian Archipelago. Environ. Biol. Fishes. 88(4): 305-309. https://doi.org/10.1007/s10641-010-9647-4

Albins, M.A., and M.A. Hixon. 2008. Invasive Indo-Pacific lionfish *Pterois volitans* reduce recruitment of Atlantic coral-reef fishes. Mar. Ecol. Prog. Ser*.* 367: 233-238. https://doi.org/10.3354/meps07620

Albins, M.A., and P.J. Lyons. 2012. Invasive red lionfish *Pterois volitans* blow directed jets of water at prey fish. Mar. Ecol. Prog. Ser. 448: 1-5. https://doi.org/10.3354/meps09580

Albins, M.A. 2013. Effects of invasive Pacific red lionfish *Pterois volitans* versus a native predator on Bahamian coral-reef fish communities. Biol Invasions. 15: 29–43. https://doi.org/10.1007/s10530-012-0266-1

Albins, M.A. 2015. Invasive Pacific lionfish *Pterois volitans* reduce abundance and species richness of native Bahamian coral-reef fishes. Mar. Ecol. Prog. Ser*.* 522: 231–243. https://doi.org/10.3354/meps11159

Anderson, L.G., J.K. Chapman, D. Escontrela, and Ch.L.A. Gough. 2017. The role of conservation volunteers in the detection, monitoring and management of invasive alien lionfish. Management of Biological Invasions 8(4): 589–598. https://doi.org/10.3391/mbi.2017.8.4.14

Anton, A., M.S. Simpson, and I. Vu. 2014. Environmental and Biotic Correlates to Lionfish Invasion Success in Bahamian Coral Reefs. PLoS ONE 9(9): e106229. doi:10.1371/journal.pone.0106229.

Arredondo-Chávez, A.T., J.A. Sánchez-Jimenez, O.G. Ávila-Morales, P. Torres-Chávez, Y. Herrerias-Diego, M. Medina-Nava, X. Madrigal-Guridi, A. Campos-Mendoza, O. Domínguez-Domínguez, and J.A. Caballero-Vázquez. 2016. Spatio-temporal variation in the diet composition of red lionfish, *Pterois volitans* (Actinopterygii: Scorpaeniformes: Scorpaenidae), in the Mexican Caribbean: insights into the ecological effect of the alien invasion. Acta Ichthyologica et Piscatoria*.* 46 (3): 185–200. doi: 10.3750/AIP2016.46.3.03

Ballew, N.G., N.M Bacheler, G.T. Kellison, and A.M. Schueller. 2016. Invasive lionfish reduce native fish abundance on a regional scale. Scientific Reports 6: 32169. https://doi.org/10.1038/srep32169

Betancur-R, R., A.P. Hines, A. Acero, G. Ortí, A.E. Wilbur, and D.W. Freshwater. 2011. Reconstructing the lionfish invasion: insights into Greater Caribbean biogeography. Journal of Biogeography. 38: 1281–1293 https://www.researchgate.net/publication/229988897.

Bogdanoff, A.K., J. Mostowy, J. Peake, C.A. Layman, A. Brito, C. Gonzalez, N. Hernández, D.T. Martinez, M.R. Bravo, and J.A. Morris. 2018. A brief description of invasive lionfish (*Pterois* spp*.*) diet composition in the Arrecifes de Cozumel National Park**.** Food Webs 17. https://doi.org/10.1016/j.fooweb.2018.e00104

Bogdanoff, A.K., C.A. Layman, J. Fleming, J.L. Akins, and J.A. Morris. 2020b. The use of artificial structure to attract and aggregate invasive lionfish (Pterois spp.) to enhance capture for control and commercialization.

Bogdanoff, A.K., C.A. Layman, T. Rezek, D.P. Nowacek, and J.A. Morris. 2020a. Lionfish (*Pterois* spp*.)* vocalizations and the potential for using passive acoustics to monitor invasive populations in the temperate and tropical western Atlantic.

Bogdanoff, A.K., K.W. Shertzer, C.A. Layman, J.K. Chapman, M.L. Fruitema, J. Solomon, J. Sabattis, S.J. Green, and J.A. Morris. 2021. Optimum lionfish yield: A non-traditional management concept for invasive lionfish (*Pterois* spp.) fisheries. Biol Invasions 23: 795-810. http://doi.org/10.1007/s10530-020-02398-z

Burford Reiskind M.O., E.M.X. Reed, A. Elias, J.J. Giacomini, A.F. McNear, J. Nieuwsma, G.A. Parker, R.B. Roberts, R.E. Rossi, C.N. Stephenson, J.L. Stevens, B.E. Williams. 2019. The genomics of invasion: characterization of red lionfish (*Pterois volitans*) populations from the native and introduced Ranges. Biol Invasions. <https://doi.org/10.1007/s10530-019-01992-0>.

Bustos-Montes, D., M. Wolff, A. Sanjuan-Muñoz, and A. Acero. 2020. Growth parameters of the invasive lionfish (*Pterois volitans*) in the Colombian Caribbean. Regional Studies in Marine Science. <https://doi.org/10.1016/j.rsma.2020.101362>.

Chagaris, D. D., Patterson III, W. F., Allen, M. S. 2020. Relative effects of multiple stressors on reef food webs in the Northern Gulf of Mexico revealed via ecosystem modeling. Frontiers in Marine Science, 7, 513. doi: 10.3389/fmars.2020.00513

Chel-Guerrero, L., D. Cua-Aguayo, D. Betancur-Ancona, A. Chuc-Koyoc, I. Aranda-González, and S. Gallegos-Tintoré. 2020. Antioxidant and chelating activities from Lionfish (*Pterois volitans* L.) muscle protein hydrolysates produced by in vitro digestion using pepsin and pancreatin. Emir. J. Food Agric. 32(1): 62-72. https://doi.org/10.9755/ejfa.2020.v32.i1.2060

Chen, Ch., K. Shao, and Y. Tu. 2004. Effect of thermal discharges on the Fish assemblages of a nuclear power Plant in northern Taiwan. Journal of Marine Science and Technology 12(5): 404-410. doi: 10.51400/2709-6998.2261. https://jmstt.ntou.edu.tw/journal/vol12/iss5/6

Chevalier-Monteagudo, P.P. 2017. Efecto de las poblaciones de *Pterois volitans* (Pisces: Scorpaenidae) sobre sus principales presas y competidores entre los peces de arrecifes en varias localidades en Cuba. Ph.D. thesis. Acuario Nacional de Cuba, La Habana, Cuba. 117 p.

Chevalier-Monteagudo, P.P., E. Gutiérrez, D. Ibarzabal, S. Romero, V. Isla, E. Hernández. 2008. Primer registro de *Pterois volitans* (Pisces: Scorpaenidae) para aguas cubanas. Solenodon 7: 37-40.

Cobián-Rojas, D., P.P. Chevalier- Monteagudo, J.J. Schimitter-Soto, R.I. Corrada- Wong, H. Salvat-Torres, E. Cabrera-Sansón, A. García-Rodríguez, A. Fernández-Osorio, L. Espinosa-Pantoja, D. Cabrera-Guerra, L.M. Pantoja-Echevaria, H. Caballero-Aragón, and S. Perera-Valderrama. 2016. Density, size, biomass, and diet of lionfish in Guanahacabibes National Park, western Cuba. Aquatic Biology 24: 219-226. https://doi.org/10.3354/ab00651

Cobián-Rojas, D., J.J. Schmitter-Soto, A. Aguilar-Perera, C.M. Aguilar, M.Á. Ruiz-Zárate, G. González, P.P. Chevalier, A. García, R. Herrera, S. Perera, H. Caballero, E. Guardia. 2018a. Diversidad de las comunidades de peces en dos áreas marinas protegidas del Caribe y su relación con el pez león. Rev. Biol. Trop. 66(1): 189-203. <http://dx.doi.org/10.15517/rbt.v66i1.28197>

Cohen, A.S., and A.J. Olek. 1989. An extract of lionfish (*Pterois volitans*) spine tissue contains acethylcholine and a toxin that affects neuromuscular-transmission. Toxicon 27(12): 1367-1376. <https://doi.org/10.1016/0041-0101(89)90068-8>

Côté, I.M., and A. Maljkovic. 2010. Predation rates of Indo-Pacific lionfish on Bahamian coral reefs. Mar. Ecol. Prog. Ser. 404: 219-225. https://doi.org/10.3354/meps08458

Cruz-López, C., A. Sanz-Ochotorena, Y. Rodríguez-Gómez, R. Lara-Martínez, M. de L. Segura-Valdéz, L.F. Jiménez-García. 2020. Morfología de las gónadas maduras del pez león (*Pterois volitans*: Pisces, Scorpaenidae) en el litoral oeste de La Habana. Rev. Invest. Mar. 40 (2): 26-42.

Cure, K., C.E. Benkwitt, T.L. Kindinger, E.A. Pickering, T.J. Pusack, J.L. Mcilwain, and M. Hixon. 2012. Comparative behavior of red lionfish *Pterois volitans* on native Pacific versus invaded Atlantic coral reefs. Mar. Ecol. Prog. Ser. 467: 181-192. https://doi.org/10.3354/meps09942

Curtis, J.S., K.R. Wall, M.A. Albins, and C.D. Stallings. 2017. Diet shifts in a native mesopredator across a range of invasive lionfish biomass. Mar. Ecol. Prog. Ser. 573: 215-228. https://doi.org/10.3354/meps12164

Dahl, K.A., and W.F. Patterson. 2020. Movement, home range, and depredation of invasive lionfish revealed by fine-scale acoustic telemetry in the northern Gulf of Mexico. Mar Biol 167: 1-22. https://doi.org/10.1007/s00227-020-03728-4

Dahl, K.A., W.F. Patterson III, A. Robertson, and A.C. Ortmann. 2017. DNA barcoding significantly improves resolution of invasive lionfish diet in the northern Gulf of Mexico. Biol Invasions. doi: 10.1007/s10530-017-1407-3.

Dahl, K.A., and W.F. Patterson III. 2014. Habitat-specific density and diet of rapidly expanding invasive red lionfish, *Pterois volitans*, populations in the Northern Gulf of Mexico. PLoS ONE 9(8): e105852. doi: 10.1371/journal.pone.0105852.

Dahl, K.A., M.A. Edwards, and W.F. Patterson III. 2019. Density-dependent condition and growth of invasive lionfish in the northern Gulf of Mexico. Mar. Ecol. Prog. Ser. 623: 145–159. https://doi.org/10.3354/meps13028

Darling, E.S., S.J. Green, J.K. O’Leary, and I.M. Côté. 2011. Indo-Pacific lionfish are larger and more abundant on invaded reefs: a comparison of Kenyan and Bahamian lionfish populations. Biol Invasions 13: 2045–2051. https://doi.org/10.1007/s10530-011-0020-0

De León, R., K. Vane, P. Bertuol, V.C. Chamberland, F. Simal, E. Imms, M.J.A. Vermeij. 2013. Effectiveness of lionfish removal efforts in the southern Caribbean. Endang Species Res. 22: 175–182. https://doi.org/10.3354/esr00542

Eaton, L., K.A. Sloman, R.W. Wilson, A.B. Gill, A.R. Harborne. 2016. Non-consumptive effects of native and invasive predators on juvenile Caribbean parrotfish. Environ. Biol. Fishes. 99 (5): 499–508. https://doi.org/10.1007/s10641-016-0486-9

Eddy, C. 2019. Trophic discrimination factors for invasive lionfish (*Pterois volitans* and *P. miles*) in Bermuda. Biol Invasions. <https://doi.org/10.1007/s10530-019-02078-7>.

Eddy, C., J.M. Pitt, J. Larkum, M.A. Altabet, and D. Bernal. 2020. Stable Isotope Ecology of Invasive Lionfish (*Pterois volitans* and *P. miles*) in Bermuda. Front. Mar. Sci.7: 435. doi: 10.3389/fmars.2020.00435

Elise, S., I. Urbina-Barreto, H. Boadas-Gil, M. Galindo-Vivas, and M. Kulbicki. 2014. No detectable effect of lionfish (*Pterois volitans* and *P. miles*) invasion on a healthy reef fish assemblage in Archipelago Los Roques National Park, Venezuela. Mar Biol. doi: 10.1007/s00227-014-2571-y

Ferreira, C.E.L., O.J. Luiz, S.R. Floeter, M.B. Lucena, M.C. Barbosa, C.R. Rocha, L.A. Rocha. 2015. First Record of Invasive Lionfish (*Pterois volitans*) for the Brazilian Coast. PLoS ONE 10(4): 1-5. <https://doi.org/10.1371/journal.pone.0123002>

Fishelson, L. 1997. Experiments and observations on food consumption, growth and starvation in *Dendrochirus brachypterus* and *Pterois volitans* (Pteroinae, Scorpaenidae). Environ. Biol. Fishes. 50: 391-403. https://doi.org/10.1023/A:1007331304122

García-Rivas, M.D.C., S. Machkour-M’Rabet, G. Pérez-Lachaud, J.J. Schmitter-Soto, C. Doneys, N. St-Jean, D. Cobián, and Y. Hénaut. 2017. What are the characteristics of lionfish and other fishes that influence their association in diurnal refuges? Marine Biology Research. doi: 10.1080/17451000.2017.1314496.

Gardner, P.G., T.K. Frazer, C.A. Jacoby, and R.P.E. Yanong. 2015. Reproductive biology of invasive lionfish (*Pterois* spp.). Front. Mar. Sci.2(7): 1-10. <https://doi.org/10.3389/fmars.2015.00007>

Goodbody-Gringley, G., C. Eddy, J.M. Pitt, A.D. Chequer, and S.R. Smith. 2019. Ecological Drivers of Invasive Lionfish (*Pterois volitans* and *Pterois miles*) Distribution Across Mesophotic Reefs in Bermuda. Front. Mar. Sci. 6:258. <https://doi.org/10.3389/fmars.2019.00258>

Green, S. J., and I. M. Côté. 2009. Record densities of Indo-Pacific lionfish on Bahamian coral reefs. *Coral Reefs.* 28 (107). doi: 10.1007/s00338-008-0446-8

Green, S.J., J.L. Akins, A. Maljkovi´c, and I.M. Côté. 2012. Invasive Lionfish Drive Atlantic Coral Reef Fish Declines. PLoS One 7 (3): e32596. doi:10.1371/journal.pone.0032596

Green, S.J., J.L. Akins, and I.M. Côté. 2011. Foraging behavior and prey consumption in the Indo-Pacific lionfish on Bahamian coral reefs. Mar. Ecol. Prog. Ser. 433: 159-167. https://doi.org/10.3354/meps09208

Guardia, E.d.l., D.C. Rojas, L. Espinosa, Z. Hernández, L. García, and J.E.A. González. 2017. Distribución y abundancia del pez león *Pterois volitans* (Scorpaeniformes: Scorpaenidae) y especies nativas asociadas en el Parque Marino Cayos de San Felipe, Cuba. Rev. Biol. Trop. 65 (1): 117-125.

Guerrero, K.A., and L.A. Franco. 2008. First record of the Indo-Pacifica red lionfish *Pterois volitans* (Linnaeus, 1758) for the Dominican Republic. Aquatic Invasions 3(2): 267-268. doi: 10.3391/ai.2008.3.2.21

Guzmán-Méndez, I.A., R. Rivera-Madrid, P. Díaz-Jaimes, M.C. García-Rivas, M. Aguilar-Espinoza, and J.E. Arias-González. 2017. First genetically confirmed record of the invasive devil firefish *Pterois miles* (Bennett, 1828) in the Mexican Caribbean. Biolnvasions Records 6(2): 99-103. https://doi.org/10.3391/bir.2017.6.2.02

Hackerott, S., A. Valdivia, S.J Green, I.M. Côté, C.E. Cox, L. Akins, C.A. Layman, W.F. Precht, J.F. Bruno. 2013. Native Predators Do Not Influence Invasion Success of Pacific Lionfish on Caribbean. Reefs. PLoS ONE 8(7): e68259. doi:10.1371/journal.pone.0068259.

Hamner, R.M., D.W. Freshwater, and P.E. Whtifield. 2007. Mytochondrial cytochrome *b* analysis reveales two invasive lionfish species with strong founder effects in the western Atlantic. J. Fish. Biol. 71(B): 214-222. <https://doi.org/10.1111/j.1095-8649.2007.01575.x>

Harris H.E., W.F. Patterson III, R.N.M. Ahrens, and M.S. Allen. 2019. Detection and removal efficiency of invasive lionfish in the northern Gulf of Mexico. Fisheries Research 213: 22–32. <https://doi.org/10.1016/j.fishres.2019.01.002>

Hunt, C.L., D.A Andradi-Brown, C.J.Hudson, J. Bennett-Williams, F. Noades, J. Curtis-Quick, O.T. Lewis, and D.A. Exton. 2020. Shelter use interactions of invasive lionfish with commercially and ecologically important native invertebrates on Caribbean coral reefs. PloS ONE 15(8), e0236200. <https://doi.org/10.1371/journal.pone.0236200>

Kindinger, K.L., and M.A. Albins. 2017. Consumptive and non-consumptive effects of an invasive marine predator on native coral-reef herbivores. Biol Invasions 19: 131–146. https://doi.org/10.1007/s10530-016-1268-1

Kochzius, M., R. Soller, M.A. Khalaf, and D. Blohmb. 2003. Molecular phylogeny of the lionfish genera *Dendrochirus* and *Pterois* (Scorpaenidae, Pteroinae) based on mitochondrial DNA sequences. Molecular Phylogenetics and Evolution 28: 396–403. <https://doi.org/10.1016/S1055-7903(02)00444-X>

Kulbicki, M., J. Bates, P. Chabanet, K. Cure, E. Darling, S.R. Floeter, R. Galzin, A. Green, M. Harmelin-Viruen, M. Hixon, Y. Letourneur, T. Lison de Loma, McClonahan, J. McIlwain, G. Moutham, R. Myers, J.K. O´Leary, S. Planes, L. Vigliola, and L. Wantiez. 2012. Distributions of Indo-Pacific lionfish *Pterois* spp. in their native ranges: implications for the Atlantic Invasion. Mar. Ecol. Prog. Ser*.* 446: 189- 205. https://doi.org/10.3354/meps09442

Labastida, E., D. Cobián, Y. Hénaut, M.C. García-Rivas, P.P. Chevalier, and S. Machkour-M ́Rabet. 2015. The use of ISSR markers for species determination and a genetic study of the invasive lionfish in Guanahacabibes, Cuba. Latin American Journal of Aquatic Research 43(5): 1011-1018. <https://doi.org/10.3856/vol43-issue5-fulltext-21>

Labastida, E., S. Machkour-M’Rabet, L. Carrillo, Y. Hénaut, and D.N. Castelblanco-Martínez. 2019. Genetic structure of Mexican lionfish populations in the southwest Gulf of Mexico and the Caribbean Sea. PloS ONE 14(10), e0222997. <https://doi.org/10.1371/journal.pone.0222997>

Lyons, T.J., Q.M. Tuckett, A. Durland, and J.E. Hill. 2020. Risk screen of lionfishes, Pterois, Dendrochirus, and Parapterois, for southeastern United States coastal waters of the Gulf of Mexico and Atlantic Ocean. Biol Invasions. https://doi.org/10.1007/s10530-020-02203-x.

Maljkovic, A., and T.E. Van Leeuwen. 2008. Predation on the invasive red lionfish, *Pterois volitans* (Pisces: Scorpaenidae), by native groupers in the Bahamas. Coral Reefs 27 (501). https://doi.org/10.1007/s00338-008-0372-9

McCleery, C. 2011. A comparative study of the feeding ecology of invasive lionfish (*Pterois volitans*) in the Caribbean**.** J. Mar. Sci. 9: 38-43.

Morris, J.A., and J.L. Akins. 2009. Feeding ecology of invasive lionfish (*Pterois volitans*) in the Bahamian archipelago. Environ. Biol. Fishes. 86: 389-398. https://doi.org/10.1007/s10641-009-9538-8

Morris, J.A., K.W. Shertzer, and J.A. Rice. 2011b. A stage-based matrix population model of invasive lionfish with implications for control. Biol Invasions 13:7–12. https://doi.org/10.1007/s10530-010-9786-8

Morris, J.A., C.V. Sullivan, and J.J. Govoni. 2011a. Oogenesis and spawn formation in the invasive lionfih, *Pterois miles* and *Pterois volitans*. Scientia Marina 75(1): 147-154. http://digital.casalini.it/2478336

Murillo-Pérez, B.I., J.J. Schmitter-Soto, D. Cobián-Rojas, and R.L. Herrera-Pavón. 2021. Trophic overlap of lionfish (*Pterois volitans*) and two native predators (*Lutjanus apodus* and *Cephalopholis cruentata*) in the western Caribbean. Biota Neotropica 21(1): e20190909. <https://doi.org/10.1590/1676-0611-BN-2019-0909>

Oxley, W.G., A.M. Ayling, A.J. Cheal, and K. Osborne. 2004. Marine surveys undertaken in the Elizabeth and Middleton Reefs Marine National Nature Reserve, December 2003. Australian Institute of Marine Science, Townsville. 42p.

Pantoja, L., P.P. Chevalier, D. Cabrera, R.I. Corrada, C. Cobián, H. Caballero, A. García, R.A. Fernández. 2017. Superposición de la dieta del pez león *Pterois volitans* (Teleostei: Scorpaenidae) con la de peces nativos de nivel trófico similar en Cuba. Boletín de Investigaciones Marinas y Costeras 46 (2): 115-134. <https://doi.org/10.25268/bimc.invemar.2017.46.2.732>

Peake, J., A.K. Bogdanoff, C.A. Layman, B. Castillo, K. Reale-Munroe, J. Chapman, K. Dahl, W.F. Patterson III, C. Eddy, R.D. Ellis, M. Faletti, N. Higgs, M.A. Johnston, R.C. Muñoz, V. Sandel, J.C. Villasenor-Derbez, J.A. Morris. 2018. Feeding ecology of invasive lionfish (*Pterois volitans* and *Pterois miles*) in the temperate and tropical western Atlantic. Biol invasions 20(9): 2567-2597. https://doi.org/10.1007/s10530-018-1720-5

Ritger, A.L., C.T. Fountain, K. Bourne, J.A. Martín-Fernández, and M.E. Pierotti. 2020. Diet choice in a generalist predator, the invasive lionfish (*Pterois volitans/miles*). Journal of Experimental Marine Biology and Ecology 524: 1-7. <https://doi.org/10.1016/j.jembe.2020.151311>

Rojas-Vélez S., J. Tavera, and A. Acero. 2019. Unraveling lionfish invasion: Is *Pterois volitans* truly a morphologically novel predator in the Caribbean? Biol Invasions. https://doi.org/10.1007/s10530-019-01946-6.(012

Ruttenberg, B.I., P.J. Schofield, J.L. Akins, A. Acosta, M.W., Feeley, J. Blondeau, S.G. Smith, and J.S. Ault. 2012. Rapid invasion of Indo-Pacific lionfishes (*Pterois volitans* and *Pterois miles*) in the Florida Keys, USA: evidence from multiple pre- and post-invasion data sets. Bulletin of Marine Cience 88(4):1051–1059. <https://doi.org/10.5343/bms.2011.1108>

Sancho, G., P.R. Kingsley-Smith, J.A. Morris, C.A. Toline, V. McDonough, and S.M. Doty. 2018. Invasive lionfish (*Pterois volitans/miles*) feeding ecology in Biscayne National Park, Florida, USA. Biol Invasions. <https://doi.org/10.1007/s10530-018-1705-4>.

Sandel, V.M. 2011. El pez león (*Pterois volitans/miles* complex) en el Área de Conservación La Amistad-Caribe, Costa Rica- estado actual de la población invasiva y perspectivas para su manejo. M.Sc. thesis. Universidad Nacional Heredia, Puntarenas, Costa Rica. p. 82

Santamaria, C.A., J. Locascio, and T.M. Greenan. 2020. First report of lionfish prey from Western Florida waters as identified by DNA barcoding. PeerJ *8*, e9922. <https://doi.org/10.7717/peerj.9922>

Saunders, P.R., and P.B. Taylor. 1959. Venom of the lionfish *Pterois volitans.* American Journal of Physiology 197: 437-440. <https://doi.org/10.1152/ajplegacy.1959.197.2.437>

Schofield, P.J. 2009. Geographic extent and chronology of the invasion of non-native lionfish (*Pterois volitans* [Linnaeus 1758] and *P. miles* [Bennett 1828]) in the Western North Atlantic and Caribbean Sea. Aquatic Invasions 4(3): 473-479. doi: 10.3391/ai.2009.4.3.5

Schofield, P.J. 2010. Update on geographic spread of invasive lionfishes (*Pterois volitans* [Linnaeus, 1758] and *P. miles* [Bennett, 1828]) in the Western North Atlantic Ocean, Caribbean Sea and Gulf of Mexico. Aquatic Invasions 5(1): 117-122. doi:[10.3391/AI.2010.5.S1.024](https://doi.org/10.3391/AI.2010.5.S1.024)

Simnitt, S., L. House, S.L. Larkin, J.S. Tookes, T. Yandle. 2020. Using Markets to Control Invasive Species: Lionfish in the US Virgin Islands. Marine Resource Economics 35(4): 319-341. <https://doi.org/10.1086/710254>

Squadrone, S., P. Brizio, C. Stella, M. Mantia, L. Favaro, B. Biancani, S. Gridelli, C. Da Rugna, M.C. Abete. 2020*.* Differential Bioaccumulation of Trace Elements and Rare Earth Elements in the Muscle, Kidneys, and Liver of the Invasive Indo-Pacific Lionfish (*Pterois* spp.) from Cuba.  Biol Trace Elem Res. <https://doi.org/10.1007/s12011-019-01918-w>

Stern N., C. Jimenez, M.F. Huseyinoglu, V. Andreou, L. Hadijioannou, A. Petrou, B. Öztürk, D. Golani, S. Rothman. 2018. Constructing the genetic population demography of the invasive lionfish *Pterois miles* in the Levant Basin, Eastern Mediterranean. Mitochondrial DNA Part A. <https://doi.org/10.1080/24701394.2018.1482284>.

Tamburello, N., I.M. Côté. 2014. Movement ecology of Indo-Pacific lionfish on Caribbean coral reefs and its implications for invasion dynamics. Biol Invasions 17(6): 1639-1653. https://doi.org/10.1007/s10530-014-0822-y

Tamburello, N., B.O. Ma, and I.M. Côté. 2019. From individual movement behaviour to landscape-scale invasion dynamics and management: a case study of lionfish metapopulations. Philosophical Transactions of the Royal Society B 374: 20180057. http://dx.doi.org/10.1098/rstb.2018.0057

Tuttle, L.J. 2017. Direct and indirect effects of invasive lionfish on coral-reef cleaning mutualists. Mar. Ecol. Prog. Ser. 569: 163–172. https://doi.org/10.3354/meps12092

Valdez-Moreno, M., C. Quintal-Lizama, R. Gómez-Lozano, and M.C. García-Rivas. 2012. Monitoring an Alien Invasion: DNA Barcoding and the Identification of Lionfish and Their Prey on Coral Reefs of the Mexican Caribbean. PLoS ONE 7(6): 1-8. https://doi.org/10.1371/journal.pone.0036636

Van den Hurk, P., I. Edhlund, R. David, J.J. Hahn, M.J. McComb, E. L. Rogers, E. Pisarski, K. Chung, M. DeLorenzo. 2020. Lionfish (*Pterois volitans*) as biomonitoring species for oil pollution effects in coral reef ecosystems. Biological Sciences. https://doi.org/10.1016/j.marenvres.2020.104915

Vega, A., E. Reynaldo, A. Fernández, J. Crúz, F. Ocaña, E. Córdova. 2015. Abundancia y distribución del pez león *Pterois volitans* (Teleostei: Scorpaenidae) en el litoral turístico de Holguín, Cuba. Solenodon 12: 72-83.

Villaseñor-Derbez, J., and R. Herrera-Perez. 2014. Brief description of prey selectivity and ontogenetic changes in the diet of the invasive lionfish *Pterois volitans* (Actinopterygii, Scorpaenidae) in the Mexican Caribbean. Pan-American Journal of Aquatic Sciences 9(2): 131-135.

Whitfield, P.E., T. Gardner, S.P. Vives, M.R. Gilligan, W.R. Courtenay Jr., G. Carleton Ray, and J.A. Hare. 2002. Biological invasion of the Indo-Pacific lionfish *Pterois volitans* along the Atlantic coast of North America. Mar. Ecol. Prog. Ser. 235: 289–297. doi:10.3354/meps235289

Whitfield, P.E., J.A. Hare, A.W. David, S.L. Harter, R.C. Muñoz, and C.M. Addison. 2006. Abundance estimates of the Indo-Pacific lionfish *Pterois volitans/miles* complex in the Western North Atlantic. Biol Invasions 9: 53-64*.* https://doi.org/10.1007/s10530-006-9005-9
